# Human Mesenchymal Stromal Cells from Bone Marrow and Neonatal Sources: A Comparative In Vitro Study of Their Myelopoietic and Lymphopoietic Supportive Capacity from Hematopoietic Progenitor Cells

**DOI:** 10.3390/biomedicines14092124

**Published:** 2026-09-20

**Authors:** Ixel Luna-Cañedo, Guadalupe Rosario Fajardo-Orduña, Rosana Pelayo, Juan Carlos Balandrán, Héctor Mayani, Constantino López-Macías, Marco Alejandro Jimenez-Ochoa, Rodolfo Eduardo Pastelin-Ruiz, Juan José Montesinos, Víctor Adrián Cortés-Morales

**Affiliations:** 1 Mesenchymal Stem Cell Laboratory, Oncology Research Unit, Oncology Hospital, National Medical Center “Siglo XXI”, Instituto Mexicano del Seguro Social, Mexico City 06720, Mexico; ixel.luna@gmail.com (I.L.-C.); guadalupefajardo@hotmail.com (G.R.F.-O.); 2 Unidad de Educación e Investigación, Instituto Mexicano del Seguro Social, Mexico City 06720, Mexico; rosana.pelayo.c@gmail.com; 3 Department of Pathology, Laura and Isaac Perlmutter Cancer Center, NYU Grossman School of Medicine, New York, NY 10016, USA; jcbalandran@gmail.com; 4 Hematopoietic Stem Cell Laboratory, Oncology Research Unit, Oncology Hospital, National Medical Center “Siglo XXI”, Instituto Mexicano del Seguro Social, Mexico City 06720, Mexico; hmayaniv@prodigy.net.mx; 5 Unidad de Investigación Médica en Inmunoquímica, Unidad Médica de Alta Especialidad Hospital de Especialidades, National Medical Center “Siglo XXI”, Instituto Mexicano del Seguro Social, Mexico City 06720, Mexico; constantino.lopez135@gmail.com; 6 Unidad de Trasplante de Médula Ósea, Unidad Médica de Alta Especialidad Hospital de Especialidades, National Medical Center “Siglo XXI”, Instituto Mexicano del Seguro Social, Mexico City 06720, Mexico; mark2145@hotmail.com; 7 Unidad Médica de Alta Especialidad Hospital de Traumatología “Dr. Victorio de la Fuente Narváez”, Instituto Mexicano del Seguro Social, Mexico City 07760, Mexico; rodolfop.md@gmail.com

**Keywords:** mesenchymal stromal cells, myelopoiesis support, lymphopoiesis support, hematopoietic progenitor cells

## Abstract

**Background/Objectives**: Mesenchymal stromal cells (MSCs) isolated from neonatal sources have been proposed in clinical cell therapy to enhance hematopoietic stem cell transplantation due to their hematopoietic support capacity. Here we evaluated this capacity, particularly the lymphopoietic potential that remains poorly understood. **Methods**: In this in vitro study, MSCs obtained from bone marrow (BM), and neonatal sources such as placenta (PL), umbilical cord blood (UCB), and Wharton’s jelly (WJ) were cultured under the same conditions to compare their abilities to support the formation of myeloid (dendritic cell-like/non-classical monocyte-like) and lymphoid (NK and B cells) lineages from hematopoietic progenitor cells (HPCs). MSCs were co-cultured with CD34^+^CD38^−^ Lin^−^ HPCs in the presence or absence of exogenous cytokines; cell lineages obtained were evaluated through flow cytometry. **Results**: Our results demonstrate that similarly to BM-MSCs, MSCs from neonatal sources have potential to support the formation of dendritic cells, non-classical monocytes, NK, and B cells. However, in contrast to BM-MSCs, neonatal sources favor the generation of lymphoid populations of advanced stages of differentiation. **Conclusions**: These results indicate that MSCs from neonatal sources have increased lymphopoietic potential compared to BM-MSCs, a finding with significant implications for the selection of MSC sources in clinical trial design.

## 1. Introduction

MSCs are components of BM stroma; BM-MSCs produce molecules and cytokines required for hematopoiesis [1]. MSCs secrete C-X-C motif chemokine 12 (CXCL12), also known as stromal cell-derived factor-1 (SDF-1), N-cadherin, FMS-related tyrosine kinase 3 ligand (Flt3L), bone morphogenetic protein 4 (BMP4), and thrombopoietin (TPO), which are associated with the maintenance, quiescence, and proliferation of hematopoietic stem cells (HSCs) [2]. Stem cell factor (SCF) [3], together with CXCL12, maintains progenitor cells in an undifferentiated state [4]. In addition, IL-7 [5], IL-6, IL-8, IL-3, and granulocyte colony-stimulating factor (G-CSF) secreted by MSCs regulate the commitment and subsequent maturation of hematopoietic lineages [6].

HSCs have the ability to generate progenitor, precursor and mature cells during hematopoiesis in BM. Leukocytes are cells derived from both myeloid lineage (dendritic cells “DCs”, monocytes, macrophages, granulocytes) and lymphoid lineage (B cells, T cells, and innate lymphoid cells), which are the mediators of host immune response [7]. Although several studies have reported growth factors required to drive differentiation of HSCs toward a specific lineage ex vivo, stromal support is necessary to improve this process. Results from our research group have shown that MSCs promote the differentiation, proliferation, and expansion of HPCs [8,9].

Study of hematopoietic support capacity of MSCs is required for optimizing HSC transplantation, particularly in patients with multiple myeloma or leukemia, as we have suggested in previous publications [9]. MSCs co-administration has emerged as a safe therapeutic strategy, with documented efficacy in mitigating graft-versus-host disease (GvHD) in affected patients [10]. Similarly, it has been shown that MSCs promote HSC engraftment due to the rapid recovery of CD45^+^ leukocytes in patients, particularly when the number of transplanted HSCs/hematopoietic progenitor cells (HPCs) is low [11]. It is important to mention that rapid hematopoietic recovery is mainly observed in the myeloid lineage [12]; however, the effect on the lymphoid lineage still requires further investigation.

BM-MSCs are commonly used in clinical trials for hematopoietic recovery and the treatment for GvHD. MSCs isolated from other tissues, such as PL, UCB, and WJ, have also been evaluated in clinical trials due to their easier accessibility. Previous work by our research group has shown that MSCs derived from PL and UCB have similar abilities to promote the proliferation and expansion of myeloid progenitors and that this ability is also similar to BM-MSCs [13]. Other research groups have reported similar functions for UCB-MSCs [14], PL-MSCs, and WJ-MSCs [15]; however, their effect on promoting lymphopoiesis has not yet been studied. 

In this in vitro study, MSCs isolated from BM, PL, UCB, and WJ were cultured under the same conditions to compare their abilities to support the formation of myeloid cells (DCs/non-classical monocytes) and lymphoid cells (NK and B cells) from HPCs.

## 2. Materials and Methods

### 2.1. Isolation and Culture of MSCs

Neonatal samples, PL (*n* = 4), UCB (*n* = 4), and WJ (*n* = 4) were obtained with the informed consent of the patients and in accordance with local ethics committee at Bernardo Sepulveda, Francisco del Paso y Troncoso Hospital, Instituto Mexicano del Seguro Social and Eduardo Liceaga General Hospital [16,17]. BM samples (*n* = 4) were obtained from donors who gave their informed consent and were undergoing surgery for fractures at the UMAE “Dr. Victorio de la Fuente Narváez” traumatology hospital, Instituto Mexicano del Seguro Social. Samples were centrifuged in a density gradient to isolate mononuclear cells (MNCs). These cells were seeded in 100 mm cell culture dishes (Corning, Corning, NY, USA) and incubated with standard conditions (37 °C, 5% CO_2_). After 14 days, the non-adherent cells were removed, retaining only the adherent cells in low-glucose DMEM medium (Biowest, Nuaillé, PC, France), supplemented with 10% fetal bovine serum (FBS, Gibco, Rockville, MD, USA), penicillin-streptomycin (1X, Biowest, Nuaillé, PC, France), L-glutamine (1X, Biowest, Nuaillé, PC, France), and gentamicin (100 mg/L, Biowest, Nuaillé, PC, France). All samples were thawed from a biobank generated in passage 5–6.

### 2.2. Immunophenotype and Differentiation Capacity of MSCs

#### 2.2.1. Immunophenotype

Characterization of MSCs membrane markers was performed according to previously described protocol [16]. Following markers were evaluated by flow cytometry: PE anti-human-CD73 (Invitrogen, Carlsbad, CA, USA), APC anti-human-CD90, PE anti-human-CD105, PE anti-human-CD13, FITC anti-human-HLA-ABC, PE anti-human-HLA-DR, PE anti-human-CD45, APC anti-human-CD34, PE anti-human-CD14, and FITC anti-human-CD31 (BioLegend, San Diego, CA, USA). Data were acquired using a FACS Canto II flow cytometer (BD Biosciences, San Diego, CA, USA) and subsequently analyzed using FlowJo v10 software (BD Biosciences, San Diego, CA, USA).

#### 2.2.2. Adipogenic Differentiation Capacity

Cells were cultured in 35 mm Petri dishes at a density of 3 × 10^4^ per dish. Upon reaching 60% confluence, adipogenic differentiation medium (Gibco, Rockville, MD, USA) was added for 21 days, with medium changes twice a week. After 21 days, cytological staining with Oil Red O (Sigma-Aldrich, St. Louis, MO, USA) was performed to visualize lipid vacuoles, as previously described [16].

#### 2.2.3. Osteogenic Differentiation Capacity

MSCs were seeded at a density of 3 × 10^4^ in 35 mm Petri dishes. Once they reached 60% confluence, osteogenic differentiation medium (Gibco, Rockville, MD, USA) was added for 21 days, with medium changes twice a week. After 21 days, alkaline phosphatase activity was detected using SIGMA FAST BCIP/NBT substrate (5-Bromo-4-chloro-3indonyl phosphate/Nitro blue tetrazolium) (10 mg/350 µL) (Sigma-Aldrich, St. Louis, MO, USA).

#### 2.2.4. Chondrogenic Differentiation Capacity

MSCs were cultured at a density of 3 × 10^4^ in 35 mm Petri dishes. Once they reached 60% confluence, chondrogenic differentiation medium (Cambrex Bio Science, East Rutherford, NJ, USA) supplemented with 10 ng of transforming growth factor-beta (TGF-β) (Peprotech, Cranbury, NJ, USA) for 21 days, with medium changes every 3–4 days. At the end of the 21 days, cytological staining with alcian blue (Sigma-Aldrich, St. Louis, MO, USA) was performed as previously described [16].

### 2.3. Isolation of CD34^+^CD38^−^Lin^−^ Enriched Cells from UCB

HPCs (CD34^+^CD38^−^Lin^−^) were isolated from UCB-MNCs by negative selection using the StemStep™ kit (STEMCELL Technologies Inc., Vancouver, BC, Canada) according to the manufacturer’s instructions and as previously described [18]. UCB-MNCs were isolated by density gradient using Ficoll-Paque Plus (Sigma-Aldrich, St. Louis, MO, USA). When UCB-MNCs were obtained, they were washed twice with 10 mL of 1X PBS (Biowest, Nuaillé, PC, France) by centrifuging at 1200 rpm for 5 min. Cell pellet was suspended in RPMI medium (Biowest, Nuaillé, PC, France) supplemented with 10% FBS. Cell viability was determined using trypan blue staining and the number of nucleated cells was determined using Turk’s diluent. UCB-MNCs were cultured in 100 mm Petri dishes (Corning) at a density of 2 × 10^6^–2.5 × 10^6^ UCB-MNCs/cm^2^ and incubated at 37 °C with 5% CO_2_. 24 h after, CD34^+^CD38^−^Lin^−^ population was enriched according to the manufacturer’s instructions, 80 × 10^6^–120 × 10^6^ UCB-MNCs were resuspended in 1 mL of serum-free medium (Stem Line; Sigma-Aldrich), and then incubated with a cocktail of antibodies directed against the surface molecules CD2, CD3, CD14, CD16, CD19, CD24, CD36, CD38, CD45RA, CD56, CD66b, and glycophorin A, followed by a second incubation with a magnetic colloid (15 min at room temperature for each incubation). Cellular suspension was then placed in a magnetic column. CD34^+^CD38^−^Lin^−^ cells were collected from the negative fraction, washed and resuspended in Stem Line culture medium. Cell viability was assessed with trypan blue and the number of nucleated cells with Turk’s diluent. To assess percentage of HPCs obtained, the following markers were evaluated through flow cytometry: APC anti-human-CD34, PE anti-human-CD38, FITC anti-human-CD14, -CD16, -CD19, -CD41a, and -CD71 (BD Bioscience). Acquisitions were performed on a FACS Canto II flow cytometer, and the data were subsequently analyzed using FlowJo v10 software.

### 2.4. Co-Cultures of HPCs with MSCs

MSCs from all sources were seeded 2 days before co-culture in 96-well plates at a density of 3 × 10^4^ cells per well, then 0.6 µg/mL mitomycin C (Mitolem, Lemery, Mexico City, Mexico) was added to stop their proliferation for 24 h. 1.5 × 10^4^ HPCs (CD34^+^CD38^−^Lin^−^ enriched cells) were added per well in Stem Line medium supplemented with 10 µL/mL penicillin-streptomycin and 10 µL/mL gentamicin. A total of 2 culture systems were used. System of cytokines 1 (C1) generates the differentiation of DCs, non-classical monocytes, and natural killer (NK) cells, for a period of three weeks in the presence of IL-7 (5 ng/mL, Peprotech), SCF (5 ng/mL, Peprotech), Flt3L (1 ng/mL, Peprotech), and IL-15 (10 ng/mL, Peprotech). System of cytokines 2 (C2) was also evaluated to promote differentiation into B cells containing IL-7 (10 ng/mL), SCF (10 ng/mL), and Flt3L (5 ng/mL) for 5 weeks. The following conditions were used as controls: condition with the presence of C1 and C2 and absence of MSCs, and MSCs in the absence of C1 or C2.

### 2.5. Total Hematopoietic Cell Proliferation

To assess total cell proliferation, the number of cells harvested from each co-culture condition was determined using trypan blue exclusion. Proliferation was evaluated on day 21 for system C1 and day 35 for system C2. Proliferation was expressed as the fold change relative to the initial seeding density of 1.5 × 10^4^ HPCs.

### 2.6. Evaluation of Support Capacity for Different Leukocyte Lineages

At the end of the differentiation period (3 weeks for system C1 and 5 weeks for system C2), hematopoietic cells were harvested from co-cultures through mechanical disaggregation and washed with 1X PBS. They were then blocked with FBS for 10 min at 4°C and washed. The following antibodies were then added for 30 min to evaluate the populations generated in C1: DCs/non-classical monocytes/NK cells (APC anti-human-CD45, PE-Cy7 anti-human-CD56, FITC anti-human-CD16, Brilliant Violet anti-human-CD11b, and APC-Cy7 anti-human-CD11c, BioLegend) and in C2: B cells (APC anti-human-CD34, PE anti-human-CD45, and APC-Cy7 anti-human-CD19, BioLegend). They were then washed with 1X PBS and fixed with 1X paraformaldehyde (BD Bioscience). Acquisitions were performed on a FACS Canto II flow cytometer (BD Biosciences, San Diego, CA, USA), and the data were subsequently analyzed using FlowJo v10 software. Results were recorded as yield per input (Y/I): absolute number of cell (DCs, non-classical monocytes, NK cells or B cells) that are produced per initial CD34^+^CD38^−^Lin^−^ HPCs seeded [19,20].

### 2.7. Statistical Analysis

Data obtained were analyzed using Graph Pad Prism 7 software, using a Kruskal–Wallis test, followed by the Mann–Whitney U test, to determine whether there was a significant difference between them. Statistical significance was assumed when *p* < 0.05.

## 3. Results

### 3.1. Characterization of MSCs

**Figure 1:**
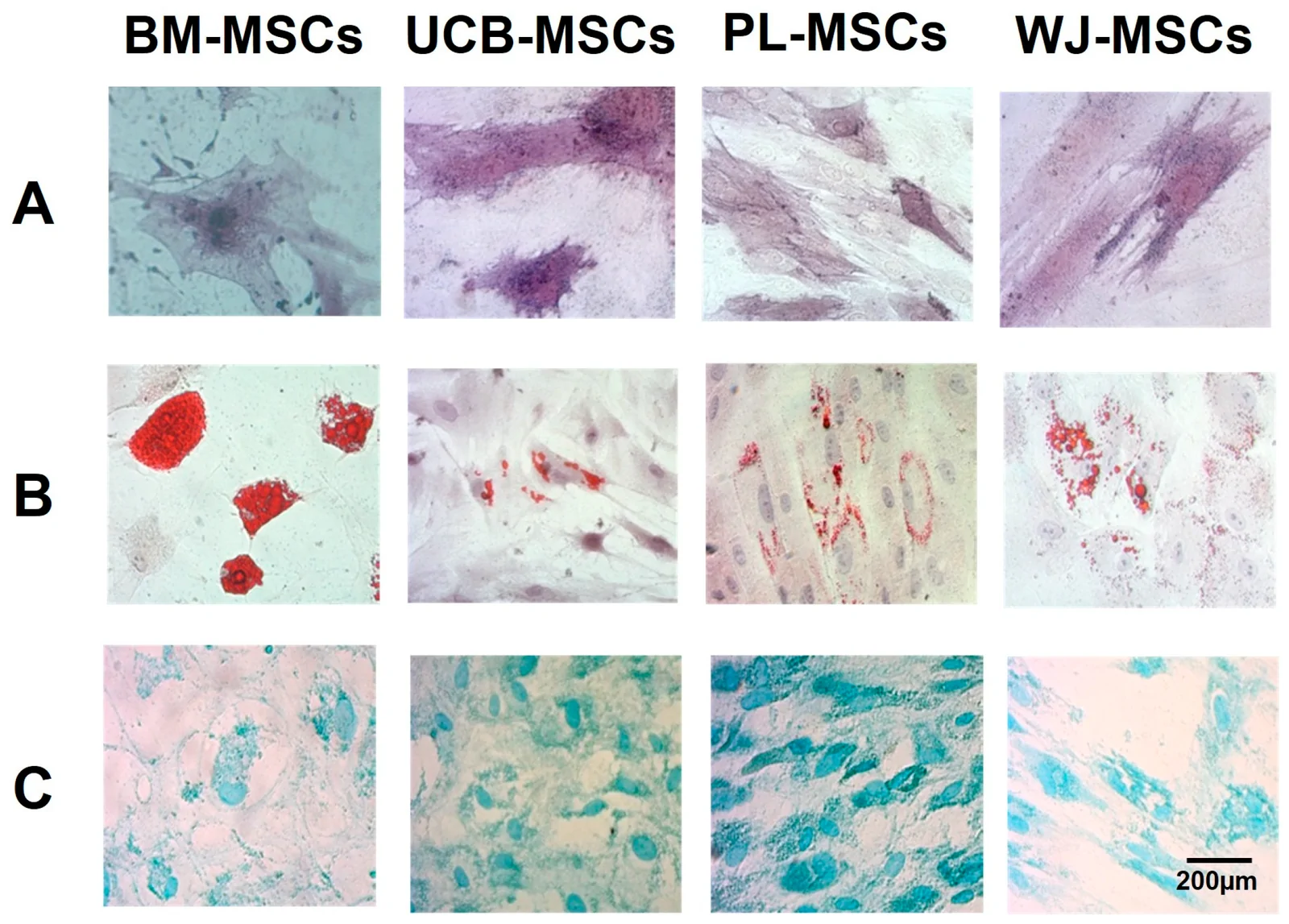
Differentiation capacity of MSCs. Representative image for: (**A**) Osteogenic differentiation evidenced by alkaline phosphatase reaction; positivity is observed in traces of alkaline phosphatase shown in purple. (**B**) Adipogenic differentiation indicated by the presence of lipid vacuoles stained with red oil dye. (**C**) Chondrogenic differentiation is indicated by the presence of chondrogenic matrix stained with Alcian blue. Size bar (200 microns).

We evaluated the phenotype of MSCs as indicated by the international society for cell and gene therapy (ISCT) [21]. We performed individual experiments with MSCs from BM (*n* = 4), UCB (*n* = 4), PL (*n* = 4), and WJ (*n* = 4) to determine characteristic membrane markers and osteogenic, adipogenic, and chondrogenic differentiation capacity. Similar to previous results [16,17], the sources were positive for the markers CD105, CD90, CD73, and CD13 and negative for the markers CD14, CD34, CD45, CD31, and HLA-DR, with no difference between the sources evaluated (Table 1). All MSC sources showed osteogenic (Figure 1A), adipogenic (Figure 1B), and chondrogenic (Figure 1C) differentiation capacity, evidenced by the presence of traces of alkaline phosphatase, lipid vacuoles, and extracellular matrix, respectively. These results indicate that MSCs evaluated fulfill ISCT criteria.

### 3.2. Initial Phenotypic Characterization of UCB-Derived HPCs

To determine the enrichment percentage of HPCs population isolated from UCB (*n* = 8), CD34^+^CD38^−^Lin^−^ phenotype was evaluated using flow cytometry. We obtained a 47% ± 24.05 enrichment of the progenitor population. In order to confirm absence of myeloid or lymphoid cells in enriched HPCs, we evaluated different subpopulations. We only observed presence of DCs at 23.95 ± 15.71, and an extremely low frequency of non-classical monocytes, NK cells, and B cells (Supplementary Table S1).

### 3.3. Capacity of MSCs to Support Leukocyte Proliferation

**Figure 2:**
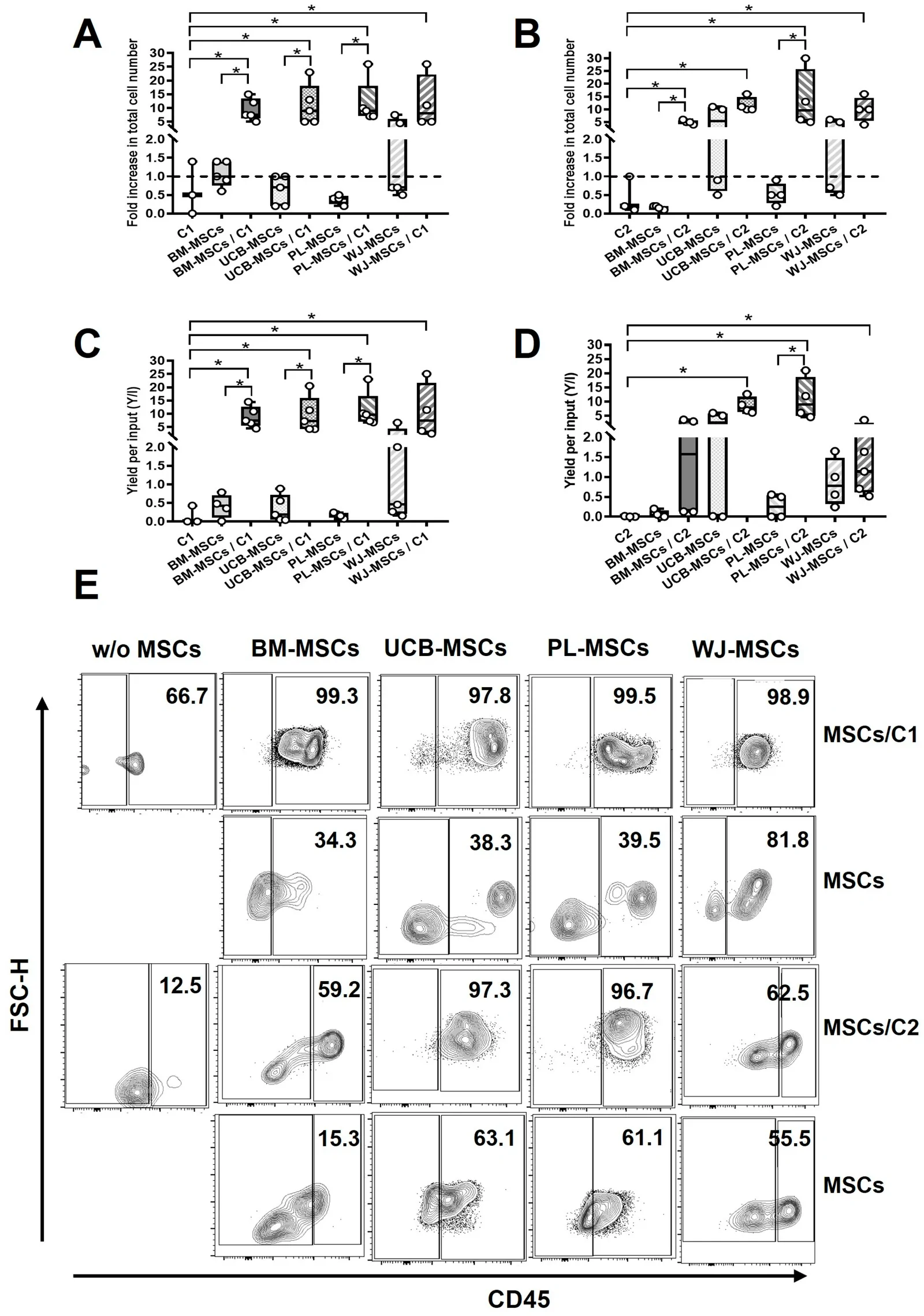
Neonatal MSCs/cytokine system promotes CD45^+^ cells expansion. Box and whisker plot show the median ± SEM of the fold increase in total hematopoietic cells generated in (**A**) C1 and (**B**) C2. Box and whisker plot shown the median ± SEM of Y/I for CD45^+^ population in C1 (**C**) and C2 (**D**). (**E**) Representative density plots of the CD45^+^ population after co-culture. Dotted line with a value of 1 corresponds to number of cells with which the co-culture was initiated. Numbers represent the percentage of CD45^+^ cells generated in each co-culture condition. * *p* < 0.05.

We previously reported that in vitro co-culture systems of MSCs with cytokines promote myelopoietic differentiation and increase the proliferation of total hematopoietic cells [9]. In our co-cultures with both cytokine systems, we found a fold increase in total cells evaluated with a hemocytometer in all MSCs sources (Figure 2A,B) (C1: BM: 9.38 ± 4.03, *p* < 0.05; UCB: 11.0 ± 7.48, *p* < 0.05; PL: 11.8 ± 8.04, *p* < 0.05 and WJ: 11.75 ± 9.91 *p* < 0.05; C2: BM: 5.0 ± 0.81, *p* < 0.05; UCB: 11.75 ± 2.87, *p* < 0.05; PL: 13.5 ± 11.56 *p* < 0.05 and WJ: 10.0 ± 4.89 *p* < 0.05) compared to HPCs in the presence of cytokines alone (C1: 0.63 ± 0.70; C2: 0.43 ± 0.49). Interestingly, when we compared condition of MSCs/cytokines with MSCs alone, we observed a decrease in total cells in BM (C1: 1.07 ± 0.39; C2: 0.15 ± 0.05), UCB (C1: 0.62 ± 0.40; C2: 5.60 ± 5.67) and PL (C1: 0.37 ± 0.12; C2: 0.52 ± 0.28), effect not observed in WJ-MSCs.

When we evaluated the Y/I of CD45^+^ population, a protein that indicates the presence of leukocytes, we found no difference among different sources of MSCs when C1 was evaluated. On the other hand, we found an increase in Y/I in all sources (BM: 8.75 ± 3.96, *p* < 0.05; UCB: 9.52 ± 6.77, *p* < 0.05; PL: 11.38 ± 6.66, *p* < 0.05 and WJ: 10.65 ± 10.38 *p* < 0.05) compared to HPCs in the presence of C1 alone (0.14 ± 0.24); similarly, when comparing the condition of MSCs/C1 with MSCs alone, a decrease in BM (0.40 ± 0.32), UCB (0.34 ± 0.36) and PL (0.16 ± 0.07) was observed, an effect that we did not find in WJ (Figure 2C,E). Furthermore, when we evaluated C2, we found that neonatal MSCs, PL (10.89 ± 7.46, *p* < 0.05), UCB (8.65 ± 2.92, *p* < 0.05) and WJ (1.50 ± 1.20, *p* < 0.05), Y/I were higher compared to C2 alone; interestingly, BM-MSCs did not increase the population in the C2 system and only PL-MSCs/C2 showed an increase Y/I in CD45^+^ population compared to MSC in the absence of cytokines (Figure 2D,E).

Our results confirm that presence of both MSCs and cytokines is required to increase the proliferation of CD45^+^ cells. On the other hand, under a system that favors myeloid/lymphoid cell differentiation (C1), all sources of MSCs have the potential to increase cell numbers; however, neonatal MSCs favor proliferation in a system that favors only the lymphoid lineage (C2).

### 3.4. Myelopoietic Support Capacity of MSCs: DCs and Non-Classical Monocytes

We previously reported that BM-MSCs, UCB-MSCs and PL-MSCs support colony-forming cells (CFCs) of monocytes, granulocytes, and monocytes/granulocytes from HPCs [9,13]; however, generation of myeloid populations—DCs-like (CD45^+^CD11b^+^CD11c^+^CD16^−^) and non-classical monocyte-like (CD45^+^CD11b^+^CD11c^+^CD16^+^) cells—has not been evaluated in in vitro systems.

**Figure 3:**
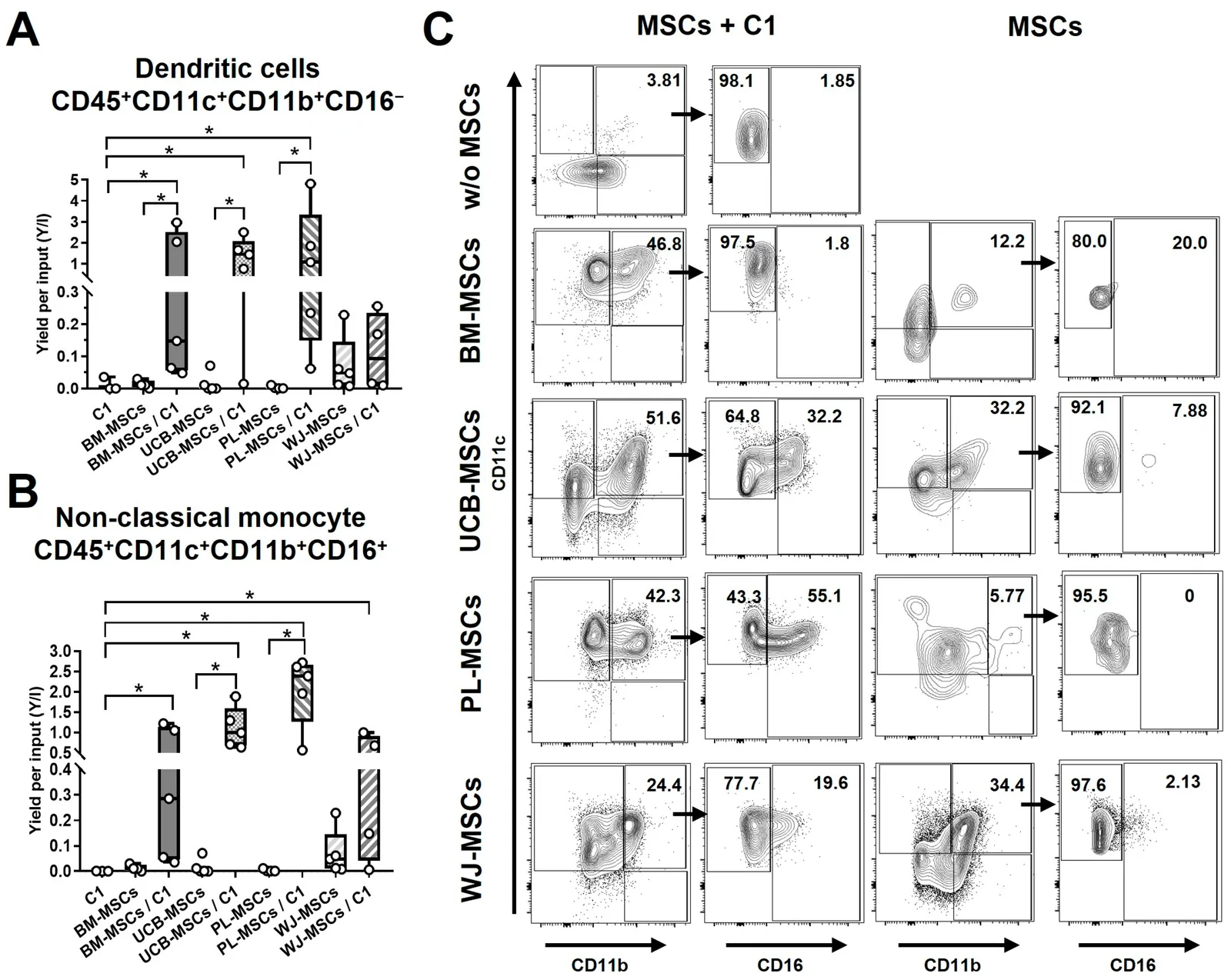
Neonatal MSCs support myelopoiesis of dendritic cells-like and non-classical monocyte-like from HPCs. Box and whisker plot shows median ± SEM of Y/I for the generation of (**A**) dendritic cells-like and (**B**) non-classical monocytes-like. (**C**) Representative density plots after 3 weeks of co-culture with C1 (IL-7, SCF, Flt3L e IL-15); numbers indicate the percentage of subpopulations that express CD11c/CD11b and CD11c/CD16 (CD16^+^ and CD16^−^); arrow indicates the precursor gate used to evaluate the expression of CD11c/CD16. * *p* < 0.05.

In MSCs/C1, when we evaluated DCs Y/I, we found an increase in BM (1.05 ± 1.36, *p* < 0.05), UCB (1.27 ± 0.94, *p* < 0.05), and PL (1.60 ± 1.92, *p* < 0.05), compared to conditions with MSCs without cytokines or HPCs in the absence of MSCs, no differences found with WJ-MSCs (Figure 3A,C).

When we evaluated non-classical monocytes Y/I, UCB-MSCs/C1 (1.10 ± 0.50, *p* < 0.05), and PL-MSCs/C1 (2.05 ± 0.87, *p* < 0.05) increased compared to HPCs in the absence of MSCs and MSCs in the absence of C1. Interestingly, BM-MSCs/C1 (0.53 ± 0.50, *p* < 0.05) and WJ-MSCs/C1 (0.45 ± 0.40, *p* < 0.05) only increased Y/I compared to HPCs/C1 (Figure 3B,C).

Our insights indicate that BM-MSCs/C1, UCB-MSCs/C1, PL-MSCs/C1 and WJ-MSCs promote the generation of myelopoietic populations.

### 3.5. Lymphopoietic Support Capacity of MSCs: NK Cells

It has previously been reported that presence of a MSCs monolayer with IL-2, IL-15, IL-3, and Flt3L promotes NK cells expansion in an in vitro system [22]. However, the differentiation of NK cell-like phenotype derived from HPCs in the presence of neonatal MSCs has not been described. In our study, we evaluated the expression of CD56 and CD16, markers described for NK cells (CD56^hi^CD16^−^, CD56^hi^CD16^lo^, CD56^hi^CD16^hi^, CD56^lo^CD16^−^ y CD56^lo^CD16^+^), to verify the generation of NK cell-like phenotype from HPCs.

**Figure 4:**
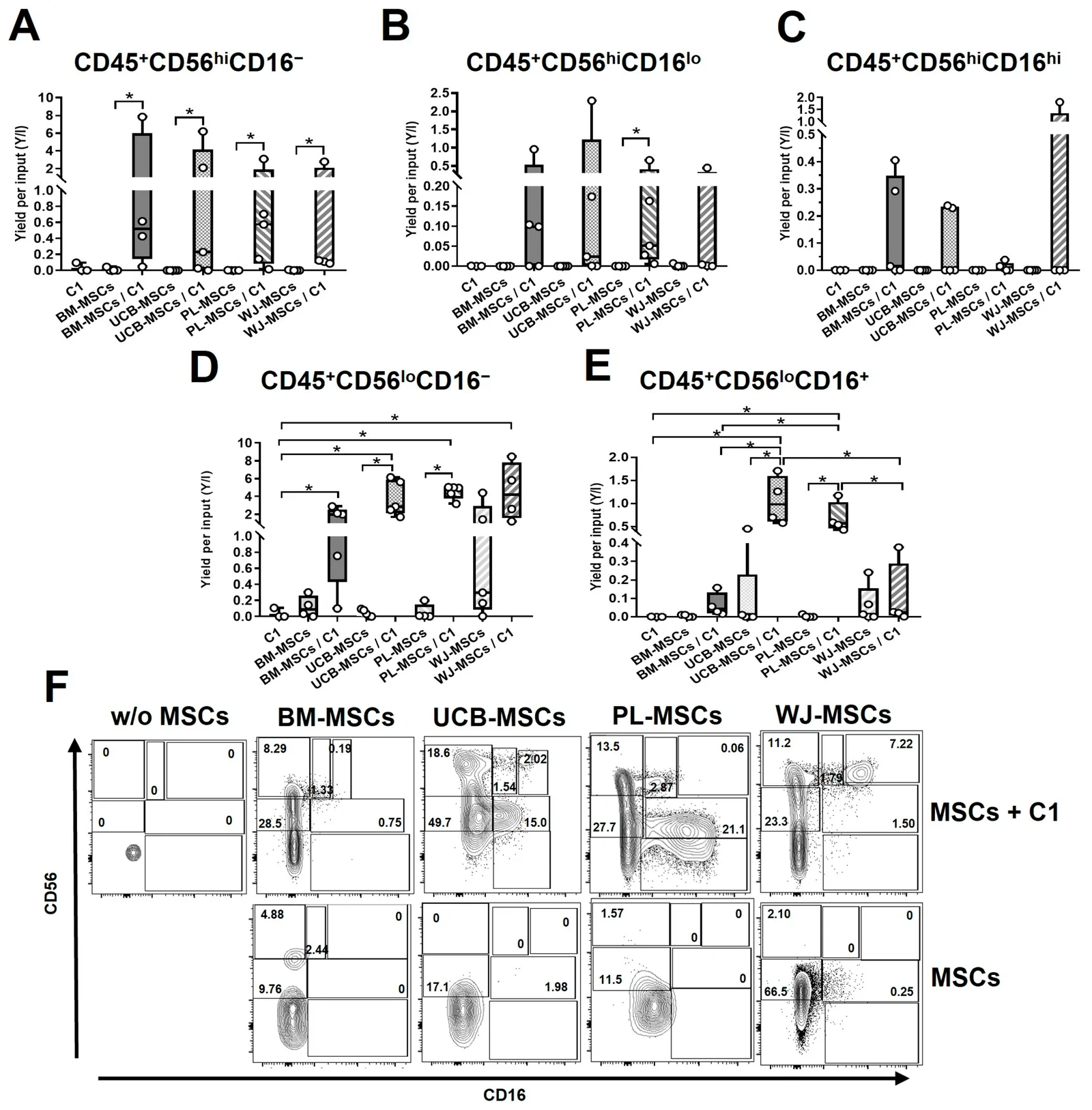
Neonatal MSCs support lymphopoiesis of NK cell-like from HPCs. Box and whisker plot shows median ± SEM of Y/I for the generation of (**A**) CD56^hi^CD16^−^, (**B**) CD56^hi^CD16^lo^, (**C**) CD56^hi^CD16^hi^, (**D**) CD56^lo^CD16^−^, (**E**) CD56^lo^CD16^+^ phenotype. (**F**) Representative density plots after 3 weeks of co-culture with C1 (IL-7, SCF, Flt3L e IL-15); numbers indicate the percentage of subpopulations that express CD56/CD16. * *p* < 0.05.

In CD56^hi^CD16^−^ population, we found that BM-MSCs/C1 (2.22 ± 3.74, *p* < 0.05), UCB-MSCs/C1 (1.71 ± 2.65, *p* < 0.05), PL-MSCs/C1 (0.90 ± 1.26, *p* < 0.05), and WJ-MSCs/C1 (0.77 ± 1.34, *p* < 0.05) increased Y/I compared to MSCs in the absence of cytokines (Figure 4A,F). On the other hand, in CD56^hi^CD16^lo^ phenotype, only PL-MSCs/C1 (0.18 ± 0.150, *p* < 0.05) increased Y/I compared to PL-MSCs (Figure 4B,F). We found no differences in CD56^hi^CD16^hi^ (Figure 4C,F). When we assessed CD56^lo^CD16^−^ population, we observed that BM-MSCs/C1 (1.56 ± 1.12, *p* < 0.05), UCB-MSCs/C1 (3.77 ± 1.99, *p* < 0.05), PL-MSCs/C1 (4.50 ± 0.79, *p* < 0.05) and WJ-MSCs/C1 (4.52 ± 3.26, *p* < 0.05) increased Y/I compared to HPCs in the absence of MSCs, but only UCB-MSCs/C1 and PL-MSCs/C1 compared to MSCs without cytokines increased this population; interestingly, there was no difference with BM-MSCs and WJ-MSCs in the absence or presence of cytokines (Figure 4D,F). Finally, for CD56^lo^CD16^+^ phenotype, only UCB-MSCs/C1 (1.06 ± 0.52, *p* < 0.05) and PL-MSCs/C1 (0.68 ± 0.33, *p* < 0.05) increased Y/I compared to MSCs in the absence of cytokines and HPCs, BM-MSCs, and WJ-MSCs in the presence of cytokines (Figure 4E,F).

Our data indicates that both BM- and neonatal MSCs have lymphopoietic support potential, as they promote the differentiation of NK cell-like from HPCs. However, only UCB- and PL-MSCs promote the expression of CD16, a molecule associated with maturation process of NK cells.

### 3.6. Lymphopoietic Support Capacity of MSCs: B Cells

It has previously been described that BM-MSCs have B cell support capacity, demonstrating that they maintain this lineage in progenitor stages [23]. However, the capacity of MSCs derived from neonatal tissues to support B cell lymphopoiesis has not yet been described.

**Figure 5:**
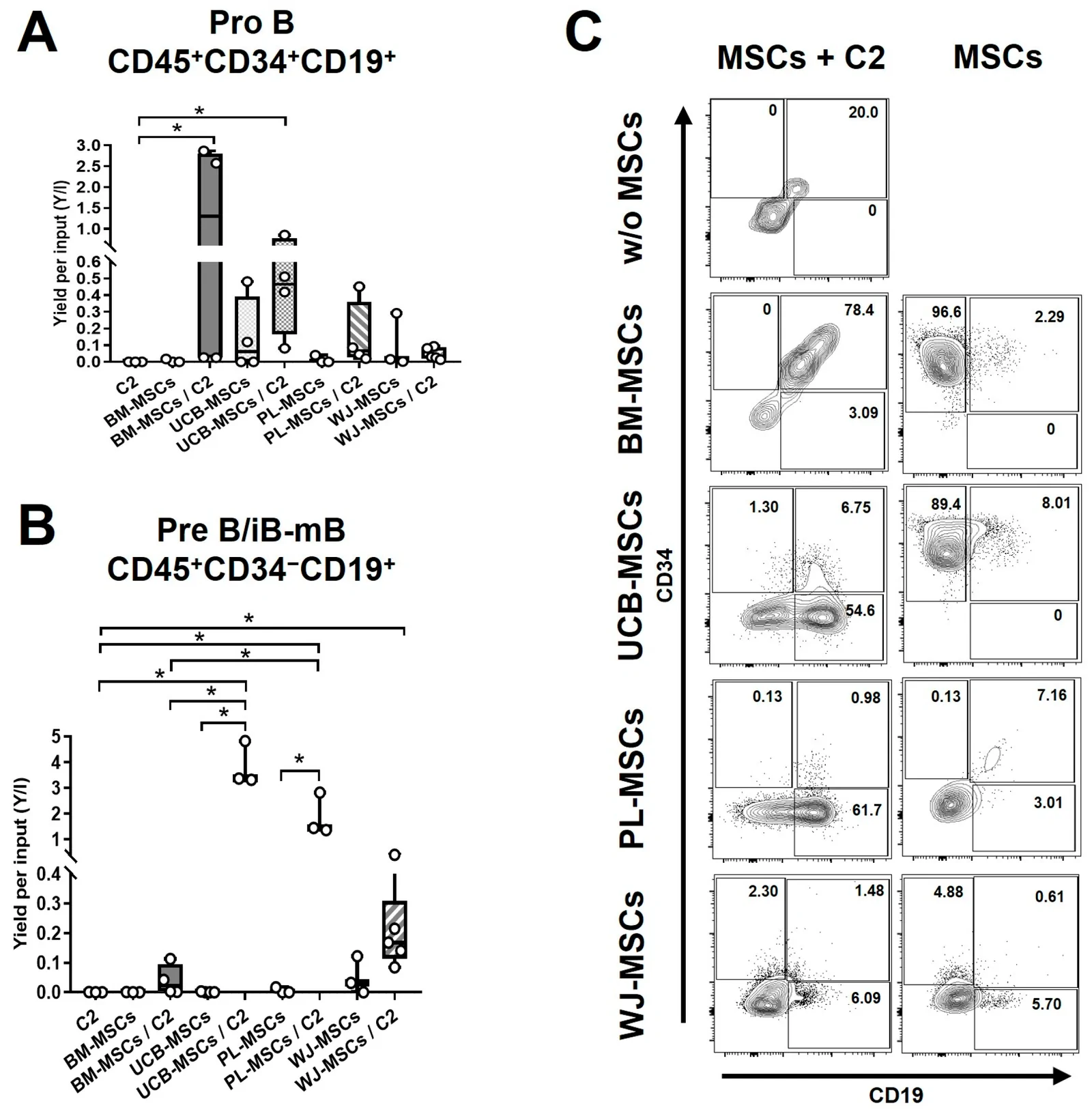
Neonatal MSCs support lymphopoiesis of B cells-like from HPCs. Box and whisker plot shows median ± SEM of Y/I for the generation of (**A**) ProB (CD34^+^CD19^+^) and (**B**) PreB/iB-mB (CD34^−^CD19^+^). (**C**) Representative density plots after 5 weeks of co-culture with C2 (IL-7, SCF and Flt3L); numbers indicate the percentage of subpopulations that express CD34/CD19. * *p* < 0.05.

To evaluate B cell differentiation C2 was used, and B cell progenitors (ProB) were identified with CD45^+^CD34^+^CD19^+^ phenotype; additionally we evaluated CD45^+^CD34^−^CD19^+^ phenotype, which describes populations of B precursors, immature B cells (iB), and mature B cells (mB), collectively referred to as PreB/iB-mB. In our results, we observed that only BM-MSCs/C2 (1.37 ± 1.36, *p* < 0.05) and UCB-MSCs/C2 (0.46 ± 0.31, *p* < 0.05) increased Y/I of ProB population compared to HPCs/C2 in the absence of MSCs (Figure 5A,C). Finally, for the PreB/iB-mB population, UCB-MSCs/C2 (3.84 ± 0.85, *p* < 0.05) and PL-MSCs/C2 (1.87 ± 0.81, *p* < 0.05) increased Y/I compared to HPCs/C2 and MSCs in the absence of cytokines; WJ-MSCs/C2 (0.20 ± 0.12, *p* < 0.05) only with HPCs; on the other hand, we did not observe an increase in PreB/iB-mB population in BM-MSCs co-culture. In addition, UCB-MSCs/C2 and PL-MSCs/C2 also increased Y/I compared to BM-MSCs/C2 (0.04 ± 0.05) (Figure 5B,C).

Our results indicate that UCB-MSCs and PL-MSCs promote B cell-like lymphopoiesis in advanced stages of differentiation, assessed by CD19 expression and loss of CD34. On the other hand, only BM- and UCB-MSCs maintain their progenitor phenotype.

## 4. Discussion

There are few studies on the generation of DCs, non-classical monocytes, or NK cells from human-HSCs/HPCs in the presence of human-MSCs. The role of BM-MSCs in B cell differentiation has been addressed; however, the impact of alternative stromal sources on lymphopoietic support remains poorly understood. For this reason, the objective of this study was to evaluate human myelopoiesis and lymphopoiesis support of different leukocyte lineages using MSCs sources from neonatal tissues (UCB, PL, and WJ) as an alternative to BM.

In our study, we show that BM-, UCB-, PL-, and WJ-MSCs express positive markers characteristic of MSCs (CD90, CD105, CD73, CD13, and HLA-ABC) and do not express hematopoietic markers (CD45, CD34, CD14, and HLA-DR) or the endothelial marker CD31. We also observed that MSCs have in vitro differentiation capacity into adipocytes, osteocytes and chondroblasts, characteristics required to define this population [21].

Previous studies indicate that proliferation and expansion of HPCs ex vivo requires the presence of stromal cells and cytokines to obtain a greater number of cells [8]. Similar to other studies, our results confirmed that the presence of MSCs/cytokines promotes increased proliferation of total hematopoietic cells in both cytokine systems, with no differences between MSC sources. These findings are consistent with other studies conducted by our group and other authors, which report the importance of a stromal support and the presence of cytokines for the expansion and proliferation of HPCs, specifically expansion of erythroid-CFCs, monocytes/macrophages-CFCs, and granulocytes-CFCs, as well as the expansion of CD34^+^CD38^−^Lin^−^ cells and long term culture-initiating cells (LTC-IC) [13,24]. HSCs expansion and proliferation is due to different molecules secreted by MSCs, among which CXCL12, SCF, Flt3L, IL-6, G-CSF, macrophage colony-stimulating factor (M-CSF), derived from BM-MSCs, UCB-MSCs, and PL-MSCs [25], as well as extracellular vesicles (EVs) containing miRNAs and different components of the TGF-β signaling chain [1], are attributed with this effect. On the other hand, it has been reported that the presence of BM-MSCs and PL-MSCs supported the expansion of CD133^+^CD34^+^ progenitors described as LTC-IC with myeloid and lymphoid potential, which express CD45 [26], as well as early lymphoid CD7^+^ progenitor cells in contact with the stroma [27]. Another study demonstrated greater lymphoid engraftment (CD3^+^ cells, CD19^+^ cells, and CD56^+^ cells) when MSCs were co-transplanted with HSCs, with the greatest effect observed when the CD271^+^ MSCs population was enriched, demonstrating the heterogeneity of this population [28]. Co-transplantation with MSCs also favored the reconstitution of naive and memory T lymphocytes [29].

We evaluated the expression of markers of DCs-like and non-classical monocyte-like cells [30] generated from HPCs in the presence of IL-7, SCF, Flt3L, IL-15, and MSCs. SCF and Flt3L are key cytokines for myelopoiesis generation, especially in progenitor stages. When evaluating CD11b and CD11c markers, all co-cultures in the presence of MSCs and cytokines generated CD11b^+^CD11c^+^ populations. CD1c^+^ dendritic cells co-express myeloid markers CD11c and CD11b and have been classified as type 2 myeloid dendritic cells (cCD2). However, it is difficult to distinguish between cDC2, monocytes, and monocyte-derived dendritic cells (mo-DCs) because they share many markers, including CD11c. Recently, combination of various markers and their expression levels have been used to distinguish them by flow cytometry. CD14 has been used to distinguish mo-DCs and CD16 to differentiate them from non-classical monocytes, which, in addition to expressing CD16, are CD11b^lo^CD11c^hi^, while mo-DCs are CD11c^+^CD14^+^CD11b^−/+^ [30]. In our study, the CD14 marker was not evaluated, but by evaluating the CD16 marker together with the CD11c and CD11b markers, we were able to identify two subpopulations in our cultures: a population with a CD11c^+^CD11b^+^CD16^−^ phenotype, which is probably common type 2 dendritic cells (cDC2)-like because they co-express CD11c and CD11b. On the other hand, CD11c^+^CD11b^+^CD16^+^ population could be CD16^+^ non-classical monocytes; however, other more specific markers would be required to determine these subpopulations. Interestingly, CD11c^+^CD11b^+^CD16^−^ subpopulation was generated with BM-, UCB-, and PL-MSCs co-culture conditions, on the other hand, we did not observe a significant difference in the generation of this population with WJ-MSCs, likely due to the limited number of evaluated samples, which restricts our statistical power; while CD11c^+^CD11b^+^CD16^+^ subpopulation was generated in all co-culture conditions in the presence of cytokines. Similar to our results, presence of BM-MSCs promotes CD11b expression in HSCs-derived DCs by Jagged1 (MSCs)/Notch (DCs) pathway, in synergy with secretion of TGF-β from MSCs [31], as well as CCL2 (MSCs)/CCR2 (HSCs) pathway [32], and these CD34^+^ cell-derived DCs have a regulatory phenotype [33]. Co-culture with MSCs promotes the expression of early markers along DCs differentiation CD117 (c-kit) and CD123 (IL3 receptor-α) [34]. Another study demonstrated that EVs secreted by MSCs promote HSCs differentiation toward myeloid progenitors by activating MyD88 [35]. On the other hand, Flt3L secreted by MSCs is required as the first step in DCs differentiation and in the presence of granulocyte-macrophage-colony-stimulating factor (GM-CSF) and SCF, CD34^+^-derived myeloid DCs are generated [36,37]. We also found that presence of MSCs generates a non-classical monocyte-like population. Literature suggests that M-CSF, also known as colony-stimulating factor 1, a molecule expressed in MSCs [38], promotes the expression of CD16 [39,40,41]; however, this mechanism still needs to be studied.

In our co-cultures, CD56^hi^ NK cell subpopulation was generated homogeneously across MSC sources; CD56^hi^ NK cells generated did not express CD16; this phenotype is mainly associated with cytokine production, although they have also been reported as precursors of the CD56^lo^CD16^+^ NK cell subpopulation with cytotoxic function, which is an advanced stage of maturation within the ontogeny of NK cells [42]. We observed an increase in CD56^lo^CD16^−^ generation in all MSC sources evaluated in the presence of cytokines; Amand and colleagues report that this population also exhibits degranulation and INF-γ production capabilities even greater than CD56^lo^CD16^+^ NKs in a challenge against K562 cells; however, functionality tests in our study are required to confirm this capacity [43]. In our system, IL-7, SCF, Flt3L, and IL-15 were used, as well as a stromal layer, which is required to increase the yield per input of CD56^+^ cells from HSCs isolated from UCB or BM [19]. We have also reported that BM-MSCs secrete CXCL12, IL-7, and IL-15 [20], molecules that participate in NK cell differentiation. It has been reported that SCF and Flt3L are necessary to regulate the first step, followed by IL-15, to achieve NK differentiation from HSCs [44]. Similar to our results, it has been reported that WJ-MSCs, in presence of IL-2, IL-15, IL-3, and Flt3L, increase generation of NK cells with decreased cytotoxic activity, as well as promote generation of precursor populations with a CD56^+^CD3^−^CD117^+^ phenotype [22]. BM-MSCs generate CD7^+^CD56^−^ early T/NK cell precursor population in the presence of Flt3L [45], without CD16 expression, which indicates a state of maturation. In our work, we found that neonatal MSCs (PL and UCB) promote CD16 expression, it has been reported that neonatal tissues support CD16^+^ NK cells with higher INF, IL-8, VEGF, and CD107a co-expression, suggesting a greater effector response capacity, which could result in better immune protection during pregnancy [46]. Also, MSCs isolated from different inflammatory conditions generate CD34^+^ cells-derived NK cells with different functional capacities [47].

Lymphopoiesis support capacity of BM-MSCs has been demonstrated to generate B cells [23]. In our findings, we found that after 5 weeks neonatal MSCs generated preB/iB-mB B lymphocytes, a population characterized by loss of CD34, but maintains expression of CD19 marker, while the BM-MSCs did not generate them. However, only BM- and UCB-MSCs maintain ProB phenotype, which indicates that BM-MSCs and UCB-MSCs promote phenotypes associated with a progenitor stage, meanwhile neonatal MSCs promote mature stages of B cell lymphopoiesis. Similar to our findings, it has been reported that BM-MSCs are capable of maintaining populations with a precursor phenotype (ProB stage), also BM-MSCs do not promote CD19 or CD3 expression, but support expansion of early lymphoid CD7^+^ progenitor cells [27] and precursor CD34^+^CD19^+^CD10^+^IgM^+^ B lymphocytes [48]. MSCs support capacity is related to Flt3L [45], IL-7, and/or components of the TGF-β superfamily (TGF-β1 and activin A) present in BM-MSCs, which have been described as negatively regulating B lymphocyte development by decreasing the expression of CD10 and CD19 in hematopoietic cells [23]. Other studies reported that factors secreted by MSCs: IL-7, TSLP, hemokinin-1 [49], CXCL12, SDF-1 [50], SCF, and FLT3-L [51], promote the phenotype and proliferation of B cell precursors.

## 5. Conclusions

This is the first study to evaluate the myelopoietic and lymphopoietic support capacity of neonatal MSCs as a possible alternative in future therapies. In our work, we report that alternative sources of MSCs have different hematopoietic support potentials. All evaluated sources of MSCs increase Y/I of CD45^+^ cells and have capacity to support the non-classical monocyte-like phenotype. Under the current experimental conditions, only WJ-MSCs did not significantly promote the generation of DC-like cells. When we evaluated support for NK cell population, while all sources have potential to favor CD56 expression in HPCs, only UCB- and PL-MSCs increased CD16 expression, a marker associated with NK maturation, which suggests a greater capacity for lymphopoiesis support for mature NK cell populations. On the other hand, for B cell support capacity, only BM- and UCB-MSCs maintain the ProB phenotype, while neonatal sources support the PreB/iB-mB phenotype, which promote CD19 expression. In summary our results indicate that neonatal MSCs have increased lymphopoietic potential to generate populations of advanced stages of differentiation, these findings have significant implications for selecting the MSC source in clinical trial design. Finally, although our study reported the myelopoietic and lymphopoietic support potential of MSCs, evaluation of new markers and functional capacity of the generated cells is necessary, as well as their evaluation by in vivo models. Further studies are currently being designed to address these questions.

## Figures and Tables

**Table 1:** Expression of MSC surface markers. Data are expressed as the mean ± standard deviation (SD) of % positive cells (BM *n* = 4; UCB *n* = 4; PL *n* = 4; WJ *n* = 4).

Marker	BM-MSCs	UCB-MSCs	PL-MSCs	WJ-MSCs
CD105	98.53 ± 6.1	92.20 ± 12.63	95.73 ± 3.09	92.25 ± 5.55
CD73	96.50 ± 2.24	97.96 ± 2.23	73.73 ± 29.79	91.18 ± 6.34
CD90	99.60 ± 0.36	74.33 ± 41.33	87.58 ± 12.21	99.23 ± 0.87
CD13	99.60 ± 0.14	97.77 ± 3.16	98.05 ± 2.33	89.88 ± 11.40
HLA-ABC	46.34 ± 26.42	62.26 ± 38.59	89.45 ± 10.10	75.15 ± 16.27
HLA-DR	0.51 ± 0.82	0.97 ± 0.87	1.35 ± 2.04	0.80 ± 0.67
CD45	0.12 ± 0.21	1.77 ± 3.15	1.36 ± 0.74	3.12 ± 4.14
CD34	0.34 ± 0.51	0.35 ± 0.62	1.62 ± 2.43	3.62 ± 3.99
CD31	0.11 ± 0.14	1.18 ± 1.63	0.68 ± 0.42	2.04 ± 1.28
CD14	0.43 ± 0.26	1.06 ± 1.84	5.1 ± 8.88	0.42 ± 0.70

## Data Availability

The original contributions presented in this study are included in the article; further inquiries can be directed to the corresponding author.

## Supplementary Materials

The following supporting information can be downloaded at https://www.mdpi.com/article/10.3390/biomedicines14092124/s1, Table S1. CD34^+^CD38^−^Lin^−^ enriched HPCs: percentage of myeloid and lymphoid cell subsets.
