# Supplementary material for: Human Mesenchymal Stromal Cells from Bone Marrow and Neonatal Sources: A Comparative In Vitro Study of Their Myelopoietic and Lymphopoietic Supportive Capacity from Hematopoietic Progenitor Cells
Source: Biomedicines. 2026 Sep 20;14(9):2124. doi: 10.3390/biomedicines14092124 (PMC13604451; doi:10.3390/biomedicines14092124)
Supplement: Supplementary file 1 [file biomedicines-14-02124-s001.zip › biomedicines-4523224-supplementary.pdf]

Supplementary Table S1. $CD34^+CD38^-Lin^-$ enriched HPCs: percentage of myeloid and lymphoid cell subsets.

**Cell population**	**% Positive cells**
Dendritic cells	
$CD45^+CD11b^+CD11c^+CD16^-$	23.95±15.71
Non classical monocytes	
$CD45^+CD11b^+CD11c^+CD16^+$	1.50±1.41
NK cells	
$CD45^+CD56^{hi}CD16^-$	0.01±0.01
$CD45^+CD56^{hi}CD16^{lo}$	0.02±0.01
$CD45^+CD56^{hi}CD16^{hi}$	0.02±0.01
$CD45^+CD56^{lo}CD16^-$	9.23±4.48
$CD45^+CD56^{lo}CD16^+$	0.07±0.05
B cells	
$CD45^+CD34^+CD19^+$	2.90±2.08
$CD45^+CD34^-CD19^+$	0.06±0.09

Values show the mean ±SD of positive cells percentage for each phenotype evaluated.
